# IL-15 Mediates Mitochondrial Activity through a PPAR*δ*-Dependent-PPAR*α*-Independent Mechanism in Skeletal Muscle Cells

**DOI:** 10.1155/2016/5465804

**Published:** 2016-09-21

**Authors:** Shantaé M. Thornton, James E. Krolopp, Marcia J. Abbott

**Affiliations:** ^1^Department of Health Science and Kinesiology, Crean College of Health and Behavioral Sciences, Chapman University, Orange, CA, USA; ^2^Department of Biological Sciences, Human and Evolutionary Biology Section, Dana and David Dornsife College of Letters, Arts and Sciences, University of Southern California, Los Angeles, CA, USA

## Abstract

Molecular mediators of metabolic processes, to increase energy expenditure, have become a focus for therapies of obesity. The discovery of cytokines secreted from the skeletal muscle (SKM), termed “myokines,” has garnered attention due to their positive effects on metabolic processes. Interleukin-15 (IL-15) is a myokine that has numerous positive metabolic effects and is linked to the PPAR family of mitochondrial regulators. Here, we aimed to determine the importance of PPAR*α* and/or PPAR*δ* as targets of IL-15 signaling. C2C12 SKM cells were differentiated for 6 days and treated every other day with IL-15 (100 ng/mL), a PPAR*α* inhibitor (GW-6471), a PPAR*δ* inhibitor (GSK-3787), or both IL-15 and the inhibitors. IL-15 increased mitochondrial activity and induced PPAR*α*, PPAR*δ*, PGC1*α*, PGC1*β*, UCP2, and Nrf1 expression. There was no effect of inhibiting PPAR*α*, in combination with IL-15, on the aforementioned mRNA levels except for PGC1*β* and Nrf1. However, with PPAR*δ* inhibition, IL-15 failed to induce the expression levels of PGC1*α*, PGC1*β*, UCP2, and Nrf1. Further, inhibition of PPAR*δ* abolished IL-15 induced increases in citrate synthase activity, ATP production, and overall mitochondrial activity. IL-15 had no effects on mitochondrial biogenesis. Our data indicates that PPAR*δ* activity is required for the beneficial metabolic effects of IL-15 signaling in SKM.

## 1. Introduction

Obesity has become a modern epidemic and is growing in both prevalence and severity throughout the world. Obesity is one of the leading causes of preventable death, with more than one-third of all Americans considered overweight or obese [1, 2]. Current treatments for obesity include a calorie restricted diet and physical exercise, but sustaining long-term weight loss has proven to be a challenge for many [3]. A mainstay for treating obesity has been to increase metabolic rate, particularly through induction of mitochondrial activity in skeletal muscle (SKM). SKM is considered the largest organ in the body and acts to carry out bodily movement through generation of ATP, primarily via mitochondrial respiration [4]. Recently, SKM has attracted attention due to its newly identified ability to release cytokines, termed “myokines,” into circulation that act to increase overall energy expenditure [5–9]. Many myokines (FGF-21, Irisin, BDNF5, interleukin-6, and interleukin-15, among others) increase in circulation following physical exercise, owing to their potential to reduce adiposity [9–11]. However, the downstream effectors of myokine signaling that positively influence metabolism remain elusive. In this regard, studies aimed at elucidating myokine signaling pathways, for the potential treatment and/or prevention of obesity, have come to the forefront of metabolic research.

Interleukin-15 (IL-15) is considered a myokine and is confirmed to increase mitochondrial activity, resulting in a decrease in overall adiposity [12–15]. Historically, IL-15 had been extensively studied as an activator of natural killer (NK) cells with antitumorigenic potential and anti-inflammatory properties [11, 16]. Interestingly, in human and rodent studies, there is evidence that circulating levels of IL-15 increase following exercise, although this notion is somewhat controversial [17–21]. It is postulated that IL-15 acts to increase glucose uptake, fatty acid oxidation, and mitochondrial activity and lipolysis to reduce adiposity [14, 22–25]. Although the evidence is clear that IL-15 possesses positive metabolic effects, its direct downstream signaling pathways remain largely unknown. Many transcriptional regulators, responsible for inducing mitochondrial dynamics, have been linked to IL-15, such as peroxisome proliferator-activated receptors (PPARs), peroxisome proliferator-activated receptor gamma coactivator 1-alpha (PGC1*α*), and silent mating type information regulation 2 homolog (SIRT1) [26–29].

PPARs are important transcriptional regulators linked to numerous beneficial metabolic effects, such as induction of mitochondrial biogenesis and fatty acid oxidation [30, 31]. Among the three isoforms, PPAR*α* is highly expressed in oxidative tissues, such as liver, heart, and type I SKM fibers, while PPAR*δ* appears to be ubiquitously expressed and acts to induce mitochondrial activity and lipid metabolism [30, 32, 33]. Both PPAR*α* and *δ* have been suggested to promote induction of mitochondrial activity in many cell types and have garnered attention as potential antiobesogenic factors [30, 34–37]. PPAR*γ* is most highly expressed in adipose tissue, both brown and white, and it plays an integral part in lipogenesis and adipogenesis [38]. The antidiabetic drug class, thiazolidinediones, acts to bind to and activate PPAR*γ* to clear circulating lipids for the restoration of insulin sensitivity [39]. Much is known regarding the positive metabolic effects of PPARs but it is not known if myokines, such as IL-15, act to upregulate their transcriptional activity [30, 36]. Interestingly, PPAR*δ* expression levels have been strongly linked to IL-15 signaling in SKM [26, 28]. However, the direct relationship between IL-15 and PPAR*δ* transcriptional activity, to modulate mitochondrial processes, has yet to be firmly established. On the other hand, there are reports that IL-15 acts to increase PPAR*α* expression levels in adipose tissue [29], but little is known regarding an IL-15-PPAR*α* relationship in SKM. Taken together, it is clear that a relationship between IL-15 and PPAR*α* and/or *δ* exits, but the depth of these relationships to induce metabolism, thereby, reducing adiposity is unknown.

Here we aimed to determine the necessity of PPAR*α* and/or *δ* as downstream mediators of the metabolically beneficial effects of IL-15 action on mitochondrial activity in SKM. Here, we show that PPAR*δ* is required for IL-15 mediated induction of mitochondrial activity independent of PPAR*α* in SKM cells.

## 2. Methods

### 2.1. Reagents

C2C12 cells were obtained from Sigma (#91031101) along with Dulbecco's Modified Eagle Medium (DMEM; #D6429), fetal bovine serum (FBS; #F0926), horse serum (#H1270), and insulin (#I9278). Recombinant IL-15 was from GenScript (#Z03309-50) and GW-6471 (#11697) and GSK-3787 (#15219) were obtained from Cayman Chemicals. Trizol (#15596), SYBR green (#A25742) and a SuperScript VILO reverse transcription kit (#11754050) were purchased from ThermoFisher. The mitochondrial dye, Mito Red, was from Santa Cruz Biotechnology (SC-#301164). Male C57BL6 mice were kept on a 12 : 12 light dark cycle and fed a standard diet and water ad libitum. Gastrocnemius muscle was obtained following euthanasia. All animal procedures were approved by the Institutional Animal Care and Use Committee at Chapman University. 

### 2.2. C2C12 Cell Culture

The mouse immortalized SKM fibroblast cell line, C2C12, was cultured in DMEM and supplemented with 10% FBS, 1% penicillin-streptomycin (10,000 U/mL), and 0.1% amphotericin B. When the cells reached 80% confluence, they were induced to differentiate into mature myotubes by supplementing the DMEM with 2% horse serum and 1 *μ*M insulin for 6 days. Myotube formation was confirmed by visualization using an inverted microscope. Upon induction of differentiation, cells were treated every other day, for 6 days, with either vehicle control (DMSO), IL-15 (100 ng/mL), 10 *μ*M of a PPAR*α* inhibitor (GW-6471), 1 *μ*M of a PPAR*δ* inhibitor (GSK-3787), IL-15 + GW-6471, IL-15 + GSK-3787, or IL-15 + GW-6471 + GSK-3787.

### 2.3. Western Blotting

Western blotting was performed as previously described [40]. Briefly, C2C12 cells were lysed in a modified RIPA buffer supplemented with protease inhibitors (Pierce). Approximately 20 *μ*g of protein from the cell homogenate preparations was separated on a 4–12% gradient gel (GenScript) via SDS-PAGE. Proteins were transferred onto Immobilon-P polyvinylidene difluoride (PVDF) membranes and blocked with 5% BSA in Tween-TBS for 1 hour. The membranes were then incubated (4°C) in 5% BSA in Tween-TBS with antibodies (1 : 1000) against PPAR*α*, PPAR*δ*, or GAPDH (Sigma). Following overnight incubation, the membranes were then probed with a secondary antibody (GenScript, 1 : 2000; or Thermo, 1 : 10,000). Blots were then washed and subjected to enhanced chemiluminescence (Pierce). Membranes were stripped in 0.5 M NaOH and probed for total proteins and subsequently GAPDH (Sigma) was used as a loading control.

### 2.4. RNA Extraction and Reverse Transcription

Standard RNA isolation procedures were performed on the cells following the 6-day treatment protocol, as previously described [41, 42]. Mouse gastrocnemius muscle was used to verify IL-2 receptor expression. Briefly, cells or muscle tissue was lysed with Trizol reagent and chloroform was added to separate the RNA from the DNA and protein fractions. RNA was precipitated from the clear phase of the Trizol-chloroform mixture, followed by centrifugation at 12,000 ×g at 4°C, with isopropanol. The RNA pellet was washed with 75% ethanol and centrifuged at 7,500 ×g for 5 minutes, at 4°C, and the pellets were air-dried. Using RNAse-free water, the pellets were resuspended and the RNA purity and concentration were quantified using a NanoDrop spectrophotometer. Reverse transcription of RNA to cDNA was performed on 2 *μ*g of RNA using SuperScript reverse transcriptase VILO kit.

### 2.5. Mitochondrial DNA Assessments

Following the IL-15 treatment protocol, genomic DNA was isolated using a miniprep DNA isolation kit (Sigma). Briefly, 5 × 10^6^ of cells in suspension, in lysis buffer, were mixed with RNase and proteinase K and incubated at 70°C for 10 minutes. Ethanol was added and then homogenates were transferred to the binding columns and subjected to a series of washes. The columns were air-dried and the DNA was eluted. DNA concentration and purity were assessed using a NanoDrop spectrophotometer (Thermo). Real time qPCR was carried out and a mitochondrial DNA marker was compared relative to a nuclear encoded marker (18S) and the corresponding sequences are displayed in Table 1.

### 2.6. Real Time Quantitative PCR

Real time qPCR was performed on the cDNA, using SYBR green in a 96-well plate. The primers are displayed in Table 1. GAPDH was used as an internal control and the ddCT method was used to calculate gene expression levels.

### 2.7. Live Cell Mitochondrial Activation Assay

Following the IL-15 treatment protocol, as described above, live cells were stained using a dye that becomes sequestered in active mitochondria [42]. The cells were then fixed with phosphate buffered formalin and DAPI was used as a nuclear stain. Fluorescence levels were assessed using an inverted Zeiss microscope and images were captured using an Axiovision camera. Relative and absolute fluorescence levels were calculated using Image J software. Measurements were corrected for total cell fluorescence to account for the variation in myotube size.

### 2.8. Citrate Synthase and ATP Assays

Citrate synthase (CS) activity was measured using a previously described protocol with some modifications [42–44]. To assess CS activity, C2C12 cell lysates were added to a 96-well plate containing 100 *μ*M 5, 5′-dithio-bis (2-nitrobenzoic acid) and 250 *μ*M acetyl-CoA. To initiate the reaction, 500 *μ*M oxaloacetate was added. The reaction was monitored in a microplate reader for 5 min at an ABS of 405. The specific activity was calculated as the absorbance rate per minute divided by the mercaptide extinction coefficient and expressed per *μ*g of protein. ATP was measured on cell lysates using a fluorometric kit in a 96-well plate (Sigma) and normalized to total protein.

### 2.9. Statistical Analysis

Data are presented as mean ± SEM and all calculations were carried out using GraphPad Prism 6. A one-way ANOVA was calculated to determine multiple comparisons with a Fisher's* post hoc* analysis. For comparisons between two groups Student's *t*-test was performed. A *P* level of 0.05 was used to determine statistical significance.

## 3. Results

### 3.1. IL-15 Induces Mitochondrial Activity in Skeletal Muscle Cells

**Figure 1:**
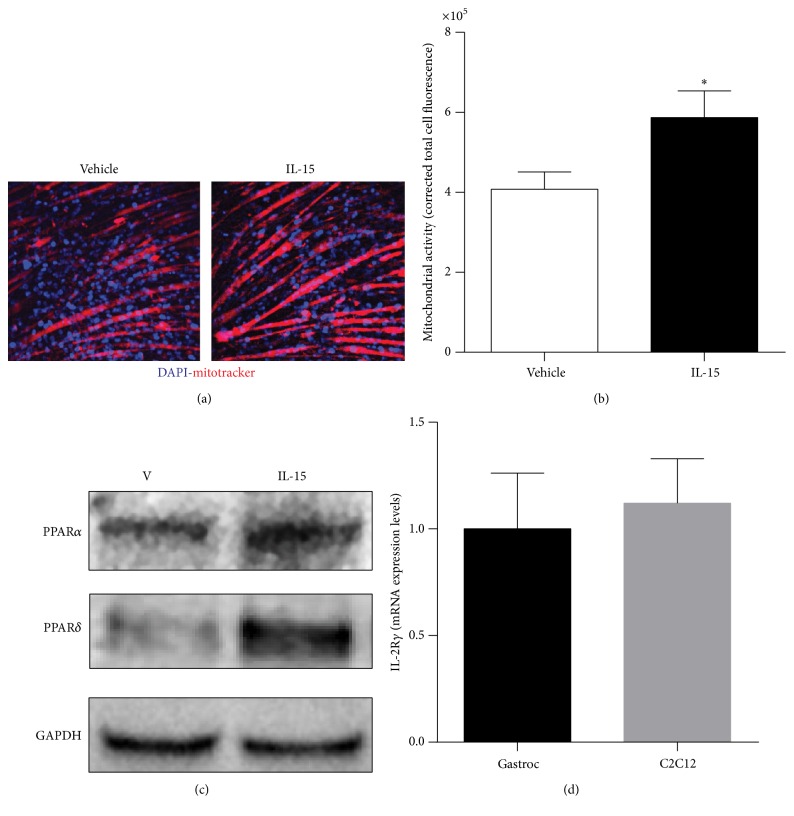
Effect of IL-15 signaling on mitochondrial activity. (a) Representative images of mitochondrial activity assessment in live cells using a fluorescent probe sequestered into active mitochondria; (b) quantifiable fluorescence corrected for myotube size; (c) western blot of PPAR*α* and PPAR*δ* protein expression; (d) mRNA expression of IL-2R*γ*. Assessments were carried out on differentiated C2C12 myotubes following treatment with 100 ng/mL of IL-15 every other day for 6 days during the differentiation protocol. Image J was used to quantify cell fluorescence. GAPDH was used as control for protein and mRNA expression assessments. All values are displayed as mean ± SEM, *n* = 3–6 per group, ^*∗*^ *P* < 0.05.

**Figure 2:**
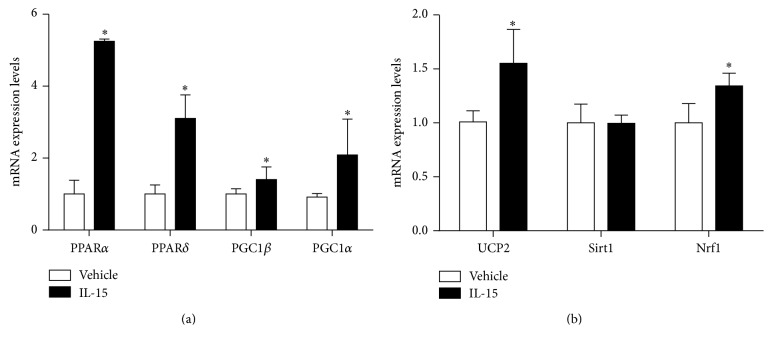
Effects of IL-15 signaling on mitochondrial associated factors. (a) mRNA expression of PPAR*α*, PPAR*δ*, PGC1*β*, and PGC1*α*; (b) mRNA expression of UCP2, SIRT1, and Nrf1. Assessments were carried out on differentiated C2C12 myotubes following treatment with 100 ng/mL of IL-15 every other day for 6 days during the differentiation protocol. GAPDH was used as an internal control for qPCR analysis. All values are displayed as means ± SEM, *n* = 6–9 per group, ^*∗*^ *P* < 0.05.

**Figure 3:**
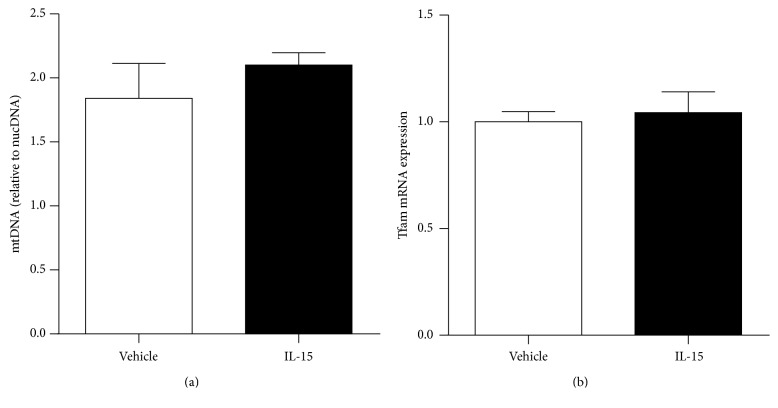
Effects of IL-15 signaling on mitochondrial biogenesis. (a) mitochondrial DNA (mtDNA) assessments; (b) mRNA expression of mitochondrial factor Tfam. Assessments were carried out on differentiated C2C12 myotubes following treatment with 100 ng/mL of IL-15 every other day for 6 days during the differentiation protocol. mtDNA was normalized to the total nuclearDNA (nucDNA) content. GAPDH was used as an internal control for qPCR analysis. All values are displayed as means ± SEM, *n* = 6.

To verify total mitochondrial activation via IL-15 signaling, a mitochondrial activity assay was performed to analyze relative mitochondrial activity in live SKM cells, as indicated by red staining (Figure 1(a)). According to quantification of the fluorescence signal, IL-15 stimulated mitochondrial activity by 44% in the SKM cells (*P* < 0.05; Figure 1(b)). IL-15 treatment stimulated increases in both PPAR*α* and PPAR*δ* protein expression levels (Figure 1(c)). To verify that the IL-15 receptor, IL-2R*γ* [45], was present in the C2C12 cells mRNA levels in mouse gastrocnemius and C2C12 cells were measured (Figure 1(d)). IL-15 induced mRNA expression levels of PPAR*α* (4-fold) and PPAR*δ* (2-fold), along with their cofactors PGC1*α* (100%) and PGC1*β* (40%), when compared to control cells (*P* < 0.05; Figure 2(a)). Owing to the capability of IL-15 to induce mitochondrial activity, the mitochondrial uncoupling protein-2 (UCP2) mRNA expression levels were increased by 50% (*P* < 0.05, Figure 2(b)). An additional factor associated with transcriptional activity of PPARs, nuclear respiratory factor 1 (Nrf1), and mRNA level was increased (30%) with IL-15 treatment (*P* < 0.05; Figure 2(b)). However, IL-15 failed to alter the expression levels of SIRT1 in the SKM cells (*P* > 0.05; Figure 2(b)). In order to determine whether IL-15 stimulated increases in mitochondrial associated factors or activity was due to increased biogenesis, we assessed mtDNA and Tfam mRNA expression levels. IL-15 had no effect on mtDNA content or on Tfam expression levels (*P* > 0.05; Figures 3(a) and 3(b)).

### 3.2. The Involvement of PPARs in IL-15 Signaling

**Figure 4:**
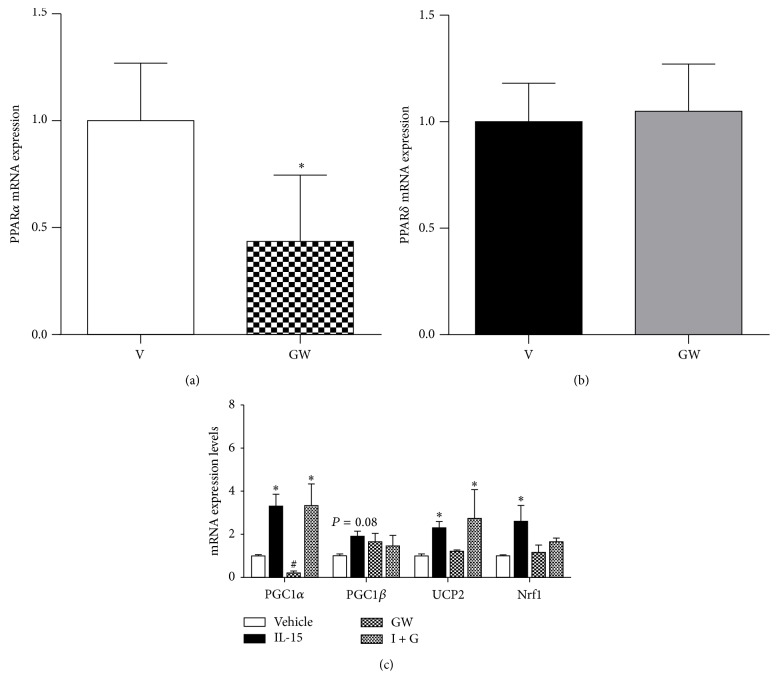
Effects of PPAR*α* inhibition on IL-15 mediated alterations of mitochondrial associated factors. (a) mRNA expression of PPAR*α* following inhibition with GW-6471 (GW); (b) mRNA expression of PPAR*δ* following exposure to GW; (c) mRNA expression of PGC1*α*, PGC1*β*, UCP2, and Nrf1 with IL-15 treatment in combination with GW. Throughout differentiation, cells were treated every other day, for 6 days, with either vehicle control (DMSO), IL-15 (100 ng/mL), 10 *μ*M of the PPAR*α* inhibitor (GW-6471), or IL-15 + GW-6471 (I + G). GAPDH was used as an internal control for qPCR analysis. All values are displayed as means ± SEM, *n* = 6–9 per group, ^*∗*^different from vehicle and GW groups; ^#^different from all groups; *P* < 0.05.

**Figure 5:**
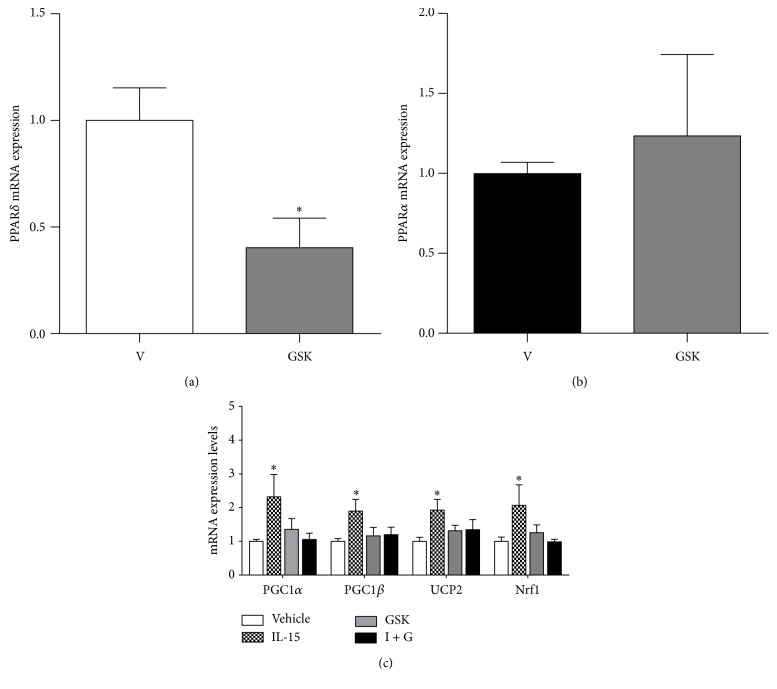
Effects of PPAR*δ* inhibition on IL-15 mediated alterations of mitochondrial associated factors. (a) mRNA expression of PPAR*δ* following inhibition with GSK-3787 (GSK); (b) mRNA expression of PPAR*α* following exposure to GSK; (c) mRNA expression of PGC1*α*, PGC1*β*, UCP2, and Nrf1 with IL-15 treatment in combination with GSK. Throughout differentiation, cells were treated every other day, for 6 days, with either vehicle control (DMSO), IL-15 (100 ng/mL), 1 *μ*M of the PPAR*δ* inhibitor (GSK-3787), or IL-15 + GSK-3787 (I + G). GAPDH was used as an internal control for qPCR analysis. All values are displayed as means ± SEM, *n* = 6–9 per group, ^*∗*^different from all groups; *P* < 0.05.

To verify the efficiency of the PPAR*α* inhibitor, GW, we confirmed a reduction in PPAR*α* mRNA expression levels by 57% when compared to vehicle control cells (*P* < 0.05; Figure 4(a)). PPAR*δ* mRNA levels were assessed to determine the specificity of GW and there were no reductions with PPAR*α* inhibition (*P* > 0.05; Figure 4(b)). Although PPAR*α* was inhibited, the stimulatory effects of IL-15 on PGC1*α* and UCP2 mRNA expression levels were maintained (*P* < 0.05; Figure 4(c)). Conversely, the IL-15 induced increases in PGC1*β* and Nrf1 mRNA levels were abolished with PPAR*α* inhibition when compared to vehicle control cells (*P* > 0.05; Figure 4(c)). Next, PPAR*δ* mRNA levels were confirmed to be reduced by 60%, when compared to vehicle control cells, with its inhibitor (GSK) (*P* < 0.05; Figure 5(a)). Conversely the PPAR*δ* inhibitor had no effects on PPAR*α* mRNA levels, confirming the specificity of GSK (*P* > 0.05; Figure 5(b)). Unlike the PPAR*α* experiments, inhibition of PPAR*δ* signaling resulted in a loss of IL-15 induced increases in PGC1*α*, PGC1*β*, UCP2, and Nrf1 when compared to vehicle control cells (*P* > 0.05, Figure 5(c)).

### 3.3. PPAR*δ* Is Required for IL-15 Mediated Increases in Mitochondrial Activity

**Figure 6:**
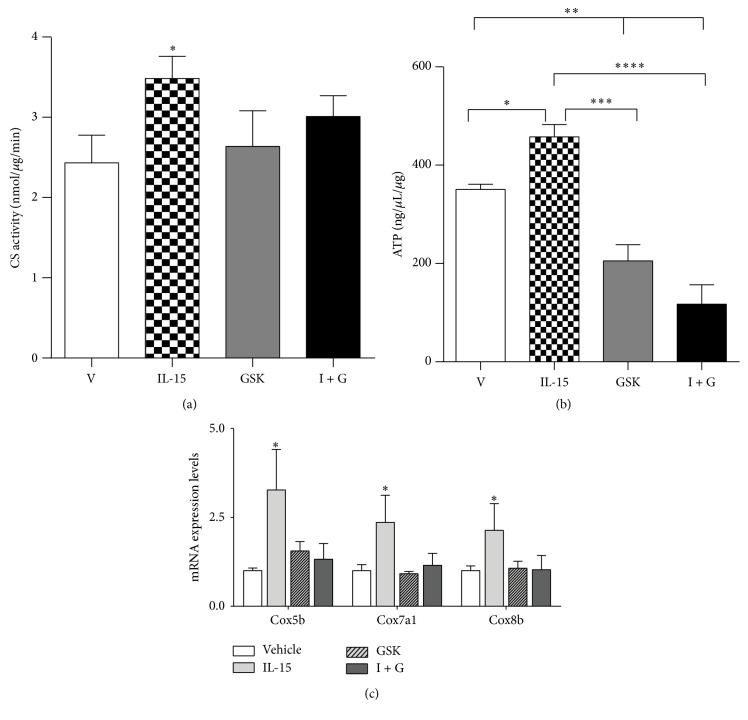
Involvement of PPAR*δ* in IL-15 mediated mitochondrial activity. (a) Citrate synthase (CS) activity; (b) total intracellular ATP content; (c) mRNA expression of cytochrome C oxidase isoforms Cox5b, Cox7a1, and Cox8b. Assessments were carried out on total cell lysates from C2C12 SKM cells following treatment every other day, for 6 days, with either vehicle control (DMSO), IL-15 (100 ng/mL), 1 *μ*M of the PPAR*δ* inhibitor (GSK-3787), or IL-15 + GSK-3787 (I + G). All values are displayed as means ± SEM, *n* = 6 per group, ^*∗*^different from vehicle control group; *P* < 0.05; ^*∗∗*^different from all groups, *P* < 0.01; ^*∗∗∗*^different from IL-15 group, *P* < 0.001; ^*∗∗∗∗*^GSK different from IL-15 group, *P* < 0.001.

**Figure 7:**
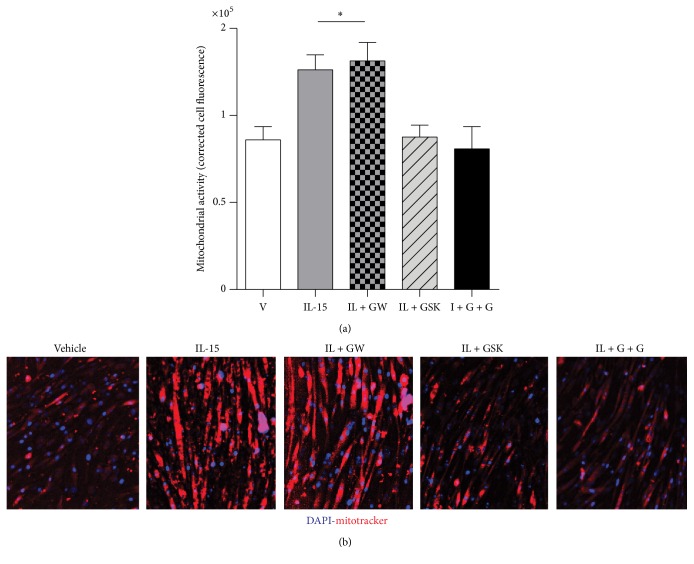
Involvement of PPAR*α* and PPAR*δ* in IL-15 mediated mitochondrial activity in live cells. (a) Quantifiable fluorescence corrected for myotube size. Assessments were carried out on differentiated C2C12 myotubes following treatment with vehicle control (DMSO), IL-15 (100 ng/mL), IL-15 + GW-6471 (I + GW), IL-15 + GSK-3787 (I + GSK), and IL-15 + GW + GSK (I + G + G) every other day for 6 days during the differentiation protocol. (b) Representative images of mitochondrial activity assessment in live cells using a fluorescent probe sequestered into active mitochondria; Image J was used to quantify cell fluorescence. All values are displayed as means ± SEM, *n* = 6 per group, ^*∗*^different from all groups; *P* < 0.05.

In the SKM cells, IL-15 stimulated increases in CS activity by 43%, when compared to vehicle cells, (*P* < 0.05) and these stimulatory effects were eliminated with PPAR*δ* inhibition (*P* > 0.05; Figure 6(a)). Likewise, ATP content was elevated (30%) with IL-15 treatment and PPAR*δ* inhibition abolished these effects (*P* < 0.05; Figure 6(b)). IL-15 induced increases in mRNA levels of cytochrome C oxidase (Cox) isoforms 5b, 7a1, and 8b were dependent on PPAR*δ* activity (*P* < 0.05; Figure 6(c)). Mitochondrial activity was assessed directly in live SKM cells and the IL-15 induced increases in mitochondrial activity remained elevated (53%), when compared to vehicle control cells, with PPAR*α* inhibition (*P* < 0.05; Figures 7(a) and 7(b)). However, inhibition of PPAR*δ* prevented the effects of IL-15 induced increases in mitochondrial activity (*P* > 0.05; Figures 7(a) and 7(b)). To further solidify the PPAR*δ*-dependent-PPAR*α*-independent IL-15 mediated signaling, mitochondrial activity was abolished with IL-15 stimulation in the presence of both PPAR*α* and PPAR*δ* inhibitors (*P* > 0.05; Figures 7(a) and 7(b)).

## 4. Discussion

Data from this study solidify the notion that IL-15 is directly involved in mediating mitochondrial activity in SKM cells. Importantly, our data indicate that PPAR*δ* activation is required for IL-15 signaling to carry out its stimulatory effects on mitochondrial activity. Further, based on our findings, we have ruled out PPAR*α* as a potential modulator of IL-15 induced mitochondrial activity. However, we have evidence for a role of an IL-15-PPAR*α* signaling relationship in mediating PGC1*β* and Nrf1 expression levels. Altogether it is clear that PPAR*δ* is required for IL-15 induced expression of mitochondrial regulators and activity in SKM cells.

In line with other reports in adipose tissue [25], treatment with IL-15 increased mitochondrial associated processes in SKM cells, as indicated by increased activity of CS and ATP production. Our data confirm other reports showing that IL-15 has the ability to induce activity of the key Krebs Cycle factor, CS, in SKM from mice [25]. It has been postulated that one route that IL-15 acts to reduce adiposity is through its ability to increase lipolysis and mitochondrial activity in adipocytes [25]. Additionally, IL-15 has been shown to increase the activity of mitochondrial processes, such as fatty acid oxidation [24, 46]. Here we show, for the first time, that IL-15 acts to directly increase overall mitochondrial activity in live SKM cells. On the other hand, it does not appear that IL-15 induces increases in mitochondrial biogenesis as indicated by the mtDNA and Tfam assessments in the C2C12 cells. Likewise, in the current study, activity of CS, ATP production, and Cox isoform expression were fully dependent on PPAR*δ* activity with IL-15 stimulation in the SKM cells. Further, it has previously been established that IL-15 increases expression levels of key factors that function to increase mitochondrial activity and biogenesis in both white and brown adipose tissue as well as in SKM [22, 26, 27, 29, 47]. Our results are in line with those findings, as PPAR*α*, PPAR*δ*, PGC1*α*, PGC1*β*, UCP2, and Nrf1 expression levels were all elevated with IL-15 stimulation. Conversely, our data do not support the notion that IL-15 stimulates mitochondrial biogenesis, which is in agreement with previously reported data in mouse SKM [48]. Unlike other studies, our treatment with IL-15 failed to increase SIRT1 expression levels [27, 28]. Here we employed an* in vitro* model to study the effects of IL-15 on SKM cells and the other reports linking IL-15 to SIRT1 were carried out in a transgenic mouse model overexpressing IL-15 [27, 28]. Therefore, it is a possibility that IL-15 induced SIRT1 expression levels are secondary to direct activation of the IL-15 signaling pathway in SKM. On the other hand, it cannot be ruled out that SIRT1 activity is regulated by IL-15, as we have only assessed mRNA expression levels. Taken together, it is clear that PPAR*δ* is required for the IL-15 induced effects on mitochondrial associated factors and activity.

Here, our data point to a strong link between IL-15 and PPAR*δ* in SKM cells, but PPAR*α* activity involvement had not been fully assessed in SKM [24, 26–28]. PPAR*α* has been associated with IL-15 signaling in adipose tissue and, with this in mind, we attempted to elucidate a potential IL-15-PPAR*α* relationship in C2C12 cells [29]. Interestingly, IL-15 induced PPAR*α* expression levels nearly 4-fold, while PPAR*δ* expression was induced only 2-fold, suggesting that PPAR*α* may be a more direct target of IL-15 signaling. However, with inhibition of PPAR*α*, PGC1*α* and UCP2 mRNA expression levels were maintained with IL-15 treatment. Conversely, Nrf1 mRNA expression levels were equivocal to the vehicle control cells with IL-15 treatment when PPAR*α* was inhibited. Nrf1 has been shown to be directly regulated by PPAR*α*, with a greater affinity than PPAR*δ*, which may explain the reduction in its expression levels with IL-15 treatment and PPAR*α* inhibition [49]. Furthermore, PGC1*β* expression levels were not statistically different from vehicle control cells with both IL-15 and PPAR*α* inhibitor. On the other hand, with PPAR*α* inhibition, the stimulatory effect of IL-15 on mitochondrial activity was maintained. Although we show a potential connection between IL-15-PPAR*α* mediated increases in some mitochondrial associated factors, these relationships do not appear to translate to functional assays, such as mitochondrial activity. It should be noted that addition of the vehicle control (DMSO) in the inhibitor studies yielded alterations in baseline mRNA expression levels when compared to mRNA levels in the absence of DMSO. Further, our data is dependent on pharmacological inhibitors of PPARs. Genetic knockdown studies would provide additional support for our data. However, the functionality of IL-15 induced increases in mitochondrial activity is relevant in mature fully differentiated SKM cells. Therefore, methodological constraints do not allow for genetic knockdown studies on fully differentiated cells with repeated treatments.

Even though our data indicate that PPAR*α* activity is not required for IL-15 mediated mitochondrial activity in SKM cells, we definitively show that PPAR*δ* activity is a requirement. Indeed, the master mitochondrial regulators, PGC1*α* and PGC1*β*, mRNA expression levels were both reduced with PPAR*δ* inhibition in combination with IL-15 stimulation. Both PGC1*α* and PGC1*β* are responsible for the numerous beneficial effects of exercise on mitochondrial processes and biogenesis and signal in concert with PPARs [50, 51]. Additionally, PGC1*α* is responsible for regulating mitochondrial uncoupling, via UCP2, and our data are in support of this pathway, as indicated by the reductions of UCP2 expression with inhibition of PPAR*δ* with IL-15 treatment [52]. The importance of IL-15 induced PPAR*δ* activation for the regulation of mitochondrial activity is further supported by the reduction of Nrf1 mRNA expression levels with PPAR*δ* inhibition and IL-15 treatment. In this regard, not only does IL-15 signal directly through PPAR*δ* but also its effects initiate a master metabolic regulation pathway, including UCP2 and Nrf1 as downstream targets.

## 5. Conclusions

It is widely accepted that PPARs play an important role in mediating mitochondrial processes to prevent and/or treat metabolic disorders [30, 32, 34, 53, 54]. We provide evidence for the requirement of PPAR*δ* as a direct target of IL-15 signaling to carry out mitochondrial processes in SKM. Additionally, our data indicate that PPAR*α* is not necessary for the beneficial effects of IL-15 signaling on mitochondrial activation in SKM. Although we have shown the importance of PPAR*δ* in IL-15 signaling, the signals directly downstream the IL-2 receptor remain unknown in SKM. Therefore, examining the effects of IL-15 on IL-2R targets such as Akt and the Jak/STAT pathway is required in SKM. In order to define the complex relationship of IL-15 signaling and PPARs further* in vivo* studies are warranted. Overall, understanding the players involved in IL-15 signaling will give rise to potential therapies for obesity and its associated disorders.

## Figures and Tables

**Table 1:** Primer sequences used for qPCR analysis.

Gene	Sequence
PPAR*α*, F	ATGGGGGTGATCGGAGGCTAATAG
PPAR*α*, R	GGGTGGCAGGAAGGGAACAGAC

PPAR*δ*, F	ACAAGGCCTCAGGGTACCA
PPAR*δ*, R	GCCGAAAGAAGCCCTTACAG

PGC1*α*, F	ACTGAGCTACCCTTGGGATG
PGC1*α*, R	TAAGGATTTCGGTGGTGACA

PGC1*β*, F	TCCTGTAAAAGCCCGGAGTAT
PGC1*β*, R	GCTCTGGTAGGGGCAGTGA

UCP2, F	CCATTGCACGAGAGGAAGGGAT
UCP2, R	GTCATGAGGTTGGCTTTCAGGAG

Nrf1, F	TTGGAACAGCAGTGGCAAGA
Nrf1, R	CTCACTTGCTGATGTATTTACTTCCAT

IL-2R*γ*, F	TACCAGACATTTGTTGTCCAGC
IL-2R*γ*, R	GCCCGTGGGATCACAAGATT

Tfam, F	CAAGTCAGCTGATGGGTATGG
Tfam, R	TTTCCCTGAGCCGAATCATCC

nucDNA, F	TTGCGATAATTATAGTGGCT
nucDNA, R	TACCTGGTTGATCCTGCCA

mtDNA, F	GGCTTTGGAAACTGACTTGT
mtDNA, R	TTGCGATAATTATAGTGGCT

Cox5b, F	GGCGGAGAAGCCCTGAA
Cox5b, R	GCTGCATCTGTGAAGAGGACAAC

Cox7a1, F	CAGCTTGTAATGGGTTCCACAGT
Cox7a1, R	CAGCGTCATGGTCAGTCTGT

Cox8b, F	AGAAAACCGTGTGGCAGAGA
Cox8b, R	GAACCATGAAGCCAACGACT

Gapdh, F	AGGTCGGTGTGAACGGATTTG
Gapdh, R	TGTAGACCATGTAGTTGAGGTCA
